# The Need and Value of Practical Demonstration and Actual Work to Acquire Skill and Success in Our Amalgam Work*Read before meeting of Toronto Dental Society, October, 1921. Printed in *Oral Health* November, 1921.

**Published:** 1921-12

**Authors:** W. E. Harper

**Affiliations:** Chicago


### THE NEED AND VALUE OF PRACTICAL DEMONSTRATION AND ACTUAL WORK TO ACQUIRE SKILL AND SUCCESS IN OUR AMALGAM WORK*

DR. W. E. HARPER, CHICAGO.

Notwithstanding the excellent qualities of the alloy that you may use, a non-leaking, strong and stable amalgam filling is dependent upon the adoption and intelligent application of a *Correct Amalgam Technic.*

Experienced and conscientious operators occasionally succeed in making an amalgam filling that will resist a leak at five to ten pounds air pressure; but within a period of one to nine months a leak generally develops at a very low air pressure.

The reasons for these failures are now known and the remedy has been at your disposal for the past eight years,

* Read before meeting of Toronto Dental Society, October, 1921. Printed in *Oral Health* November, 1921.

during which time it has stood the test of continued experimental work, clinical observation, and public demonstration, as hereinafter described; a test that has placed the Standardized Amalgam Technic beyond successful dispute by competent amalgam authority.

In accepting invitations to present this subject before many of our Dental Societies, it has been my policy to insist that interested amalgam operators in attendance make two series of test fillings.

These first series of test fillings were made preliminary to my talk and demonstration, using the amalgam procedure as carried out in every-day practice.

The second series of tests were made by the same operators, applying the Standardized Amalgam Technic (so far as it had been understood), following a two-hour talk and demonstration by myself.

The best evidence of the value of this more practical form of instruction is made significant by a comparison of the tests made "before and after taking."

I present the following report of tests made at a representative meeting. These reports may be accepted as typical of all I have attended.

During the period of my investigations immediately preceding the announcement to the profession of the importance of decided plasticity of the amalgam mass during the packing of a filling, more than 70 per cent of the test fillings made for me by experienced operators showed defects that were readily apparent to the naked eye when one of the cavity walls was removed to permit examination. Defects of this character, in every instance, are due to the condensation of a *dry mix,* a result of the forcible pinching out of the excess of mercury during the final kneading. The lack of plasticity in such a mass is manifested by a decided creaking of the amalgam during condensation. Such a mass will crack and break as it spreads, becoming so solid that it is impossible to compress it with sufficient force to eliminate these imperfections.

Upon removal of one of the cavity walls to permit examination.

GROUP 1

Twelve test fillings made as in every-day practice.

Seven fillings presented defects sufficiently large to be apparent to the naked eye. All of which leaked when the fillings were subjected to air pressure.

Four leaked under a pressure of two to four pounds.

One showed no leak at ten pounds.

GROUP 2

Twelve test fillings made by the same operators using the Standardized Amalgam Technic.

None of the fillings showed naked-eye defects.

One leaked at three pounds pressure.

Four leaked between five and eight pounds.

Seven showed no leak at ten pounds.

Ten pounds is the limit of air pressure to which I consider it proper to subject a filling immediately following its completion.

I present these as correct and beg to state that this report can be verified by any one.

When experienced operators are made to see and know the character and extent of their amalgam failures, as described, their interest is excited into the greatest possible effort to correct the fault. And, when following a two-hour talk and demonstration, so tremendous an improvement can be seen (as may be noted by a comparison of the two groups of tests), the merit of this method of instruction becomes apparent, and the value of the operative details presented by me is made conclusive.

My observations in the test work done for me have made it decidedly apparent that the reading of a paper fails to convey or explain; the rapidity of the rubbing in the mortar required to make a complete mix, also, what constitutes decided plasticity, tamping, thorough, forceful and orderly condensation. These operating details must be taught by actual demonstration followed by immediate practice.

The excuse for this paper is to urge the importance of a better understanding of these *vital points* to further urge the necessity of their conscientious application if uniformly perfect results are to be attained.

The following may be considered as a standardized amalgam technic, to be followed in the use of *ALL* high-grade alloys, all of which are slow to amalgamate and set rather quickly. Alloys that contain less that 65 per cent of silver do not belong to this class, and may be considered unreliable in some essential particular.

### PROPORTIONS

Proportions need not be exact but mercury should be plentiful, sufficient to avoid any trace of dryness in the mixing, and just within the other extreme of liquid plasticity. Either extreme is unfavorable to thorough amalgamation. When the proportions have been decided upon by a trial mix, it will save time and material to use the small amalgam balance sold for that purpose. The amount of mercury used in making a mix has nothing whatever to do with the excess mercury in the finished filling.

### MIXING

All high-grade alloys amalgamate rather slowly. It requires thorough rubbing of the particles in the presence of mercury to develop thorough amalgamation, conditions that prohibit a hand mix. Mixing is best accomplished by *rapid* rubbing in a deep mortar for a *minimum* time of *three* minutes, or a *minimum* time of one minute in the electric mixer. A measure of time is the only dependable guide to a thorough mix. At the end of the mixing period the mass should be sufficiently plastic to flatten out to about half of its diameter when rolled into a ball and dropped on the cabinet from a height of about four inches.

Thorough mixing is necessary to attain the degree of plasticity most favorable to adaption.

It is also necessary to secure the greatest strength for the filling.

An incomplete mix will be an unstable filling, by causing shrinkage or expansion in the finished filling in from one to nine months under the varying temperatures in the mouth.

The very plastic consistency described should be retained during the filling of the first two-thirds of all average large, and proximo-occlusal cavities, to insure adaption in the use of high-grade alloys. This important requirement is best accomplished by temporarily retaining sufficient of the excess mercury (commonly removed during the final mixing and kneading), to be removed ultimately, after it has served its purpose, by tamping, forceful, and orderly packing, and the final packing detail, stepping the cavity walls.

The excess mercury generally removed during the final kneading is not dangerous, because this mercury is a surplus always removed by very ordinary packing. The excess mercury that requires our special attention is that excess mercury that only becomes apparent under thorough manipulation of the amalgam mass during the insertion into the cavity.

TAMPING

Tamping has proved to be a very material aid in the removal of excess mercury, to uniform condensation, and adaption. Tamping is done by lightly tapping each piece of amalgam placed in the cavity, with the plugger, until it spreads as a uniformly thick, dense, and plastic layer, in contact with the surrounding walls and well into the angles. This procedure provides uniform bulk and plasticity under each thrust of the plugger. Tamping in conjunction with forcible and orderly condensation seems to be the simplest and the most effective means for working out the excess mercury, and experience shows a decided improvement in stability and perfection of adaption, when this operative detail is carried out.

### CONDENSATION

The packing follows tamping and should be as forceful and thorough as possible, and applied with the greatest possible uniformity. The most practical means of accomplishing this in the mouth is by an *orderly stepping* of the plugger. Following the detail of tamping, we start the packing of each piece of amalgam with a medium or large plugger at the centre of the filling, stepping a little less than its diameter in *one* direction—outward in a gradually enlarging circle until the cavity walls are reached. The surplus mercury expressed is scraped off with the side of the plugger, and this surplus may be used for the finishing of the filling, by forcibly pinching out the fluid mercury to the point where you have remaining a rather stiff but workable mass, which is again tamped and packed, repeating the operation until the cavity margins are well covered with hardened amalgam.

### STEPPING THE CAVITY WALLS

The final condensation of each piece of amalgam is done by inclining the smallest plugger toward the cavity wall and stepping about half of its long diameter (size of plugger about 1.2 by 1.8 m.m. in diameter) immediately against the walls, and well into the angles *once* around the cavity.

With an orderly packing, the excess mercury is always driven in one direction outward, where it is at all times free to come to the surface to be removed.

The necessity for packing force and thoroughness has always been taught (though not sufficiently well applied), but the need for plasticity, tamping, and orderly condensation as applied to amalgam, are new thoughts or facts that *must* be accepted, to secure uniformity in results in adaptation, strength, and stability.

A filling unequally condensed is to the same extent unequal in its resistance to crushing stress and flow, strongest where it has received the maximum condensation, weakest where it has received the minimum condensation, little or no excess mercury where the filling has received the maximum condensation, and much excess mercury where it has received the minimum condensation. Excess mercury, always means mercury *unamalgamated.*

Excess mercury or an uneven distribution of that remaining, is equally fatal to the stability of the filling when exposed to the varying temperatures in the mouth. I again repeat: tamping, thorough, forceful, and orderly condensation, are the factors that will eliminate these troubles, that have proved unsurmountable in the use of the old amalgam technic.

Packing without order results in an uneven distribution of the mercury and in mercury pockets. A thrust of the plugger on one side of the filling drives the excess mercury to the other side, and a thrust on the other side of the filling drives much of it back again, and a third thrust between the two, pockets much of the excess and forces some of the excess mercury back into the amalgam previously condensed. To successfully remove excess mercury we must keep it moving in *one direction—outward* where it is at all times free to come to the surface to be scraped off with the side of the plugger.

Excess mercury (not zinc) is almost entirely responsible for the discrediting results developed by time in our amalgam work.

Excess mercury *must* be used to make a complete mix, and *must* be retained during the packing into the cavity, to make possible airtight adaptation to cavity walls. The removal of this necessary excess mercury *must* be accomplished *during* the insertion of the mass into the cavity, and this need will be found sufficiently well provided for in the Standardized Amalgam Technic I have presented.

### THE MATRIX

The heavy packing-force required to insure the complete removal of excess mercury makes the use of the matrix imperative in all cavities in which one or more of the surrounding walls are missing. The matrix may be held by ligature, or by one of the mechanical retainers sold for that purpose. It should be held securely and should be reasonably well adapted to the tooth at all points along the cavity margins.

It is very much easier to avoid a large bulb of amalgam in the interproximate space, and an overlap at the gingival margin, than it is to trim off the excess during or after the setting of the amalgam. For this purpose, I insert a round, tapering, wood, toothpick from the lingual side to wedge the matrix solidly against the gingival margin. This wedge assists materially in preserving the interproximate space and saves much time in trimming the filling to form.

The matrix must be so held that it may be adjusted, or stretched, to permit restoration of the contact point when building the filling.

If, while making a filling, the amalgam mass becomes stiff, more mercury may be added and kneaded into the mass to secure the desired plasticity, when making large restorations, without impairing the strength of the finished filling.

It is not the per cent of mercury contained in the finished filling that is important. It is the per cent of unamalgamated mercury contained that does the damage.

Amalgam in its proper place is an indispensible filling material, the more desperate the case, the more desperate the need for its use. A non-leaking amalgam filling will never discolor tooth structure, and with the practical elimination of its most discrediting features, let us hope that we may be honored in its use, in its proper place.

I cannot permit this occasion to pass without the correcting of faults in adaption, strength, and instability in our amalgam work. Because every-day observation in the dental office brings a sadder story, common in the use of all filling materials, but much more common in the use of amalgam, namely: Failure to make restoration of the interproximate space and contact form, and neglect in the trimming of rough overhanging margins. Indeed, in many instances, no pretense is made in this direction. This neglect is more serious in its consequences than the faults in the use of amalgam I have spent the past twelve years of my professional career to correct. Because, amalgam generally saves the teeth, notwithstanding faults in adaptation, strength, and instability. But failure to restore the interproximate space and contacts, to at least a reasonable degree, means the beginning of a gingivitis, to be followed by destruction of the septal tissues and the peridental membrane, with all the possibilities of focal infection. Destruction of these tissues is irreparable, and commonly results in the loss of the tooth; in which event we may expect a general disturbance of occlusion, separation of teeth, with other serious consequences.

Overlapping and rough margins result in much trouble and the very small amount of time required to make a reasonably good finish, particularly along the gingival margin, makes this neglect absolutely unjustifiable.

When amalgam is used in its proper place, or when used in an effort to bring our service within the financial reach of our poorer patrons, I plead that we make the best possible effort, that our valuable time will permit, to avoid the serious injury and destruction to the septal tissues and peridental membrane, by giving, at least, reasonable attention to the restoration of the interproximate space and contact forms, and to the smooth finishing of the margin along the gingival. *True professional service demands this.* These are operating faults,—not faults of amalgam.

